# Involuntary Autobiographical Memories in Schizophrenia: Characteristics and Conditions of Elicitation

**DOI:** 10.3389/fpsyt.2020.567189

**Published:** 2020-10-09

**Authors:** Mélissa C. Allé, Fabrice Berna, Jean-Marie Danion, Dorthe Berntsen

**Affiliations:** ^1^Department of Psychology and Behavioral Sciences, Center on Autobiographical Memory Research, Aarhus University, Aarhus, Denmark; ^2^Inserm U1114, Strasbourg University, University Hospital of Strasbourg, Strasbourg, France

**Keywords:** diary study, involuntary autobiographical memory, schizophrenia, self-reflection, triggers

## Abstract

Involuntary autobiographical memories are mental representations of personally experienced past events that come to mind spontaneously, with no preceding attempt to recall them. They have been showed to be more frequent and more emotional in the psychosis continuum. Although schizophrenia is strongly associated with thought disorders, including cognitive intrusions of thought, images, semantic knowledge, research on patients' involuntary autobiographical memories is limited. We undertook two studies to compare involuntary and voluntary remembering in schizophrenia and the conditions in which involuntary memories occurs in those patients, both in daily life (*n* = 40), using a diary method, and in an experimental context (*n* = 50). Overall, results showed that the conditions of elicitation of involuntary memories differ in patients, as patients were more sensitive to memory triggers, especially internal triggers, in comparison to controls. Relatedly, patients' involuntary memories—mostly related to mundane events with low emotional load—were experienced more frequently. Although patients' involuntary and voluntary memories were less clear, more poorly contextualized and associated with a lower belief in occurrence than those of controls, patients considered them as more central to the self, in comparison to controls. The results are discussed in relation to patients' self-reflective impairments.

## Introduction

Involuntary autobiographical memories are mental representations of personally experienced past events that come to mind unexpectedly, that is, with no preceding attempt to recall them (1, 2). Such memories are frequent in everyday life, happening mostly during periods of inactivity and boredom (3, 4) and their occurrence is generally preceded by identifiable cues in the environment or in thought (1, 2, 5). Indeed, involuntary retrieval is based on associative processes that make a connection between the current (inner and outer) situation and features of personal memories (1). These automatic processes are beyond cognitive control (6). The conceptual contrast to involuntary autobiographical memories is voluntary autobiographical memories—that is, memories of events that are the result of a consciously and deliberately initiated retrieval process.

Everyday involuntary autobiographical memories have been described as functional in different ways [see (7)]. For example, such memories allow us to effortlessly mentally navigate between past and future, which is considered to be a major evolutionary advantage (8, 9). In addition, involuntary episodic remembering is central to the regulation of behavior through the knowledge transfer from a past to a present situation (i.e., from the remembered event to the situation in which the memory arises). Indeed, at times, an involuntary memory may instigate a direct change in an ongoing activity or suggest a solution to a problem and thus hold a directive function (10, 11).

Although normally adaptive, or at least benign, involuntary autobiographical remembering can be a source of distress. This is most clearly observed in relation to post-traumatic stress disorder (PTSD). In PTSD, the traumatic event is “persistently re-experienced” in terms of, among other things, “recurrent and intrusive distressing recollections of the event” (12).

In schizophrenia, thought intrusion (i.e., repetitive and unwanted thoughts, images, or impulses interrupting ongoing activity) (13, 14), is very common and has been related to psychotic symptoms and hallucination (15). More precisely, people with schizophrenia experiencing hallucinations also experience more anxiety-related and depression-related intrusive thoughts than both individuals with or without psychiatric disorders (16). People with schizophrenia found these intrusions more distressing, less controllable and tended to feel sadder as a result of their intrusions in comparison to both control groups (16).

In addition, patients with schizophrenia experience mind-pops—that is, fragments of semantic knowledge (words, songs, phrases, or images) coming to mind unexpectedly—more frequently than depressed or non-clinical controls (17, 18). Particularly, patients in the study by Elua et al. (18) reported significantly more verbal and visual mind-pops than controls, but the groups did not differ in the number of musical mind-pops. Patients' mind-pops were also more associated with negative content than those of controls. In this study, almost half of the mind-pops were reported with no apparent triggers, which can make them seem “uncontrollable,” a key characteristic of hallucination as well (19, 20).

With regard to involuntary autobiographical memory *per se*, very few studies have investigated them throughout the psychosis continuum (which includes psychotic symptoms from subclinical manifestations to the clinically significant ones typically observed in individuals diagnosed with a psychiatric illness) (21).

Jones and Steel (22) investigated the propensity of involuntary retrieval in individuals with schizotypy (i.e., a personality trait related to psychosis) (23) and showed that individuals scoring high on schizotypy reported more involuntary autobiographical memory in a free-association word task than those who scored low. Most of the memories reported had a neutral or positive content, for all participants.

In the same population, Holmes and Steel (24) showed that individuals with high schizotypy scores reported more trauma-related intrusions after watching a trauma film, in comparison to individuals with low schizotypy scores. The authors emphasized the parallel between intrusions in PTSD and some psychotic disorders. Relatedly, people diagnosed with schizophrenia and comorbid PTSD have more involuntary autobiographical memories in comparison to people with schizophrenia without PTSD (25). This increase of involuntary autobiographical memory frequency was not associated with psychotic symptoms severity (25).

Following up upon some of these earlier studies, we showed previously (26) that both neutral and trauma-related involuntary autobiographical memories occur more frequently in the daily life of individuals with attenuated psychotic symptoms [that is, individuals who are part of the so-called “psychosis continuum” reflecting a continuum encompassing a full range of psychotic symptom expressions from subclinical to clinical disorders, see (21)]. Importantly, we also observed that involuntary autobiographical memories and future projections were robustly related to hallucination proneness in the general population (27). This relationship was consistent even when controlling for a wide range of other factors known to be associated with hallucination, such as thought intrusion, rumination, executive functioning, depressive, and dissociative symptoms, personality traits, and mental imagery (27).

Moreover, individuals with attenuated psychotic symptoms experienced their involuntary memories as more vivid and intrusive, and associated with a stronger feeling of reliving, in comparison to control individuals (26). In addition, involuntary autobiographical memories were more emotionally intense, had greater negative mood impact and were associated with negative content, such as past traumatic experiences, in individuals with attenuated psychotic symptoms compared to control participants. These results suggested enhanced emotional responses associated with involuntary autobiographical memory in individuals with attenuated psychotic symptoms.

Importantly, these studies on involuntary autobiographical memories in psychosis were conducted with individuals in the subclinical end of the psychosis continuum and did not involve patients diagnosed with schizophrenia (the extremity of the psychosis continuum). Thus, in spite of the overall importance of involuntary autobiographical memories in daily life cognition (2) and their potential role on hallucination symptoms (27), little is known about involuntary autobiographical memory in schizophrenia.

In contrast, substantial work on voluntary retrieval of autobiographical memories has been conducted in this population. Patients with schizophrenia exhibit consistent and severe autobiographical memory impairments when those memories are strategically and voluntarily retrieved, in response to specific instructions or sensory cues (28, 29). Recently, we compared voluntary vs. involuntary retrievals of autobiographical memories in schizophrenia, in order to disentangle the role of retrieval deficits in patients' autobiographical memory impairments (30). This work consistently showed that involuntary and voluntary autobiographical memories were similarly impaired in schizophrenia, ruling out the hypothesis that autobiographical memory impairment is caused by patients' problems with self-initiated voluntary retrieval.

Here we report two studies that pursued a thorough investigation of the context in which involuntary autobiographical memories arise in schizophrenia, their mechanisms of activation, but also the content of patients' involuntary autobiographical memories and some of their qualitative characteristics. Both studies were designed to compare involuntary and voluntary remembering in schizophrenia and to provide convergent evidence using two different research methodologies.

Study 1 employed an Ecological Momentary Assessment (EMA); which is an ecological method that involves the repeated collection of real-time data on subjects' behavior and experience in their natural environments (i.e., involuntary autobiographical memories in this context). This methodology allowed us to collect critical information, using a diary, about the contexts in which involuntary memories arise in people with schizophrenia in the course of their everyday lives, which no other method can supply. Importantly, only very few studies have used this methodology in individuals diagnosed with schizophrenia (18, 31, 32) and none have used it in relation to involuntary autobiographical memory, as it is challenging to run such protocols in patients presenting with cognitive disorders and motivation impairments.

Study 2 was designed to examine involuntary and voluntary autobiographical memories in individuals with schizophrenia, using a new experimental set up developed especially for this purpose. Study 1 had high ecological validity, but was time-consuming and cognitively-demanding for patients, whereas Study 2 enabled memory assessment in a controlled experimental setting, and in ways that were easier for the patients to accomplish.

Both studies examined a theoretically motivated selection of variables exploring the mechanisms of activation of involuntary autobiographical memories (including the attentional state preceding the memory, type of triggers and the participants' sensitivity to triggers), as well as memory content and memory qualities (including emotion, mood impact, intrusiveness, feeling of control, belief in occurrence, and me-ness). These variables were selected on the basis of previous research on involuntary autobiographical memories in healthy populations [e.g., (2, 33)] as well as previous research on voluntary autobiographical memory in schizophrenia [e.g., (28, 34, 35)].

Despite the exploratory nature of the present studies, some hypotheses were made based on the literature on spontaneous thoughts and involuntary memories in the psychosis continuum. First, based on thought intrusion research (16), we hypothesized that involuntary autobiographical memories of patients would be associated with emotional impairments, in comparison to their voluntary memories as well as compared with both involuntary and voluntary memories of controls. Second, we hypothesized that patients' involuntary memories would also be associated with a higher feeling of intrusiveness and a lower feeling of control (16), compared to their voluntary memories as well as compared with both involuntary and voluntary memories of controls. Third, we hypothesized that patients with schizophrenia would experience involuntary memories more frequently than control participants (18, 22, 25, 26).

## Study 1

### Method

#### Participants

Thirty outpatients with schizophrenia (8 women and 22 men) from the Psychiatry Department of Strasbourg University Hospitals (France) took part in the study. They met the DSM-5 (12) criteria for schizophrenia spectrum disorder (patients with schizophrenia, *n* = 25; patients with schizoaffective disorder, *n* = 5) and, in the preceding 3 months, they had not experienced any change in their symptoms or medication, or been hospitalized. Symptoms of schizophrenia were assessed by a psychiatrist using the Positive and Negative Syndrome Scale (36). All patients were receiving long-term, second-generation antipsychotic treatment. A group of 23 healthy control participants was matched to patients with respect to age, gender, and level of schooling (see Table 1). None of the control participants had a psychiatric illness or was taking medication. No participants from either group had a history of neurological disorders or substance abuse. Control participants who scored higher than 8 on the Beck Depression Inventory (37) and patients scoring higher than 4 on the Calgary Depression Scale for Schizophrenia (38) were excluded from the study. The Strasbourg Ethics Committee approved the study (IC-RCB: 2017-A00316-47), and all participants gave their written, informed consent after the procedures had been fully explained to them. All participants who completed the study were compensated for their participation.

**Table 1:** Patients with schizophrenia and control participants' demographic information and clinical measures for study 1 (*n* = 40) and study 2 (*n* = 50).

	**Patients with Schizophrenia^a^**		**Control Participants^a^**		**Statistics**		
	***M***	***SD***	***M***	***SD***	***t*_**(38)**_**	***d***	**95% IC**
**Study 1**							
Age (years)	38.15	9.96	37.10	10.06	0.33	0.10	[−5.35, 7.46]
Level of education	12.40	1.79	12.80	1.88	−0.69	−0.22	[−0.40, −1.57]
CDSS	1.42	1.24	–	–	–	–	–
BDI	–	–	1.35	0.91	–	–	–
PANSS—Positive symptoms	13.41	3.43	–	–	–	–	–
PANSS—Negative symptoms	15.59	6.15	–	–	–	–	–
PANSS—General	23.76	6.71	–	–	–	–	–
PANSS—Total	52.82	13.43	–	–	–	–	–
Length of illness	12.40	7.73	–	–	–	–	–
**Study 2**							
Age (years)	38.20	9.67	38.68	10.39	−0.17	−0.05	[−6.19, 5.23]
Level of education	11.64	2.21	12.08	1.87	−0.76	−0.22	[−1.60, 0.72]
CDSS	1.72	1.55	–	–	–	–	–
BDI	–	–	1.87	1.12	–	–	–
PANSS—Positive symptoms	14.5	4.93	–	–	–	–	–
PANSS—Negative symptoms	19.18	6.63	–	–	–	–	–
PANSS—General	27.04	6.86	–	–	–	–	–
PANSS—Total	55.67	21.76	–	–	–	–	–
Length of illness	14.58	7.15	–	–	–	–	–

*BDI, Beck Depression Inventory; CDSS, Calgary Depression Scale for Schizophrenia; PANSS, Positive and Negative Syndrom Scale*.

a *n = 20 for Study 1 and n = 25 for Study 2*.

#### Design

The design was a 2 (Group: individuals with schizophrenia vs. control participants) ×2 (Retrieval: involuntary vs. voluntary) mixed design with Retrieval as within-subject variable.

### Materials

#### Memory Diary

A well-established ecological momentary assessment method of collecting involuntary and voluntary autobiographical memories was employed in the current study (39). Participants were instructed to record 15 involuntary and 15 voluntary memories in a diary over the course of several days and to self-rate the characteristics of their memories. As mentioned in the introduction, some measures of memory, related to executive functions (i.e., specificity, reliving, vividness, context, coherence), are reported in Allé et al. (30), which addresses the role of executive deficit in autobiographical memory impairment in schizophrenia.

##### Involuntary autobiographical memory collection

The first step consisted in recording involuntary memories in a small notebook that was to be carried at all times. Participants were instructed to record information about the first two memories that occurred spontaneously (without preceding attempt to recall the memory) on a given day. Immediately after a memory had occurred, participants were asked to write down brief details of the memory and to answer questions assessing the characteristics of the current situation in which the autobiographical memory popped-up (e.g., location, ongoing action, ongoing thinking and memory triggers), using five-point Likert scales (see Table 2 for details). There was no requirement to record involuntary memories every day.

**Table 2:** Questions included in the memory questionnaires.

**Common measures to Study 1^¤^ and Study 2^£^**	
Memory description^*^	Please describe the memory. *Open answer*
Me-ness	To which extend do you consider this memory as yours? *From 1 = I have the feeling that this memory is not mine to 5/7 = I am convinced that this memory is mine, I lived this event*
Spontaneous rehearsal	This memory has previously come to me “out of the blue,” without me trying to think about it. *From 1 = Not at all to 5/7 = Very Often*
Visual perspective	What is your location in the memory? *From 1 = Looking out from my own eyes to 5/7 = I could see myself in the memory like an external observer*
Belief in occurrence	I believe the event really occurred in the way I remember it, I haven't imagined or created anything that did not occur. *From 1 = 100% imaginary to 5/7 = 100% real*
Emotional valence	Was the memory particularly emotional? *From 1 = Very negative to 5/7 = Very positive*
Mood impact	Did the memory affect your mood? *Better/Worse/No impact*
Physical reaction	While remembering the event, I had a physical reaction (I laughed, felt tense, sweaty, felt cramps or butterflies in my stomach, my heart pound or race, etc.). *From 1 = Not at all to 5/7 = Extremely strong*
Temporal distance	(Study 1) How old is the memory? *Days/weeks/months/years* (Study 2) How old were you when the event occured? *Open answer*
**Additional measures—Study 1**	
Location^*^	Where were you, when the memory came to you?
Ongoing action^*^	What were you doing?
Ongoing thinking^*^	Were you thinking of something else simultaneously? *Yes/No* If yes, please describe what
Trigger^*^	Compare the content of the memory with what had taken place in your thoughts and surroundings right before the memory came to your mind. Did anything in your surroundings, or anything in your activity, attention, or what had been on your mind repeat itself to the memory? Check the most salient commonalities: *People/Places/Sensory experiences/Objects/Feelings/Life theme/Activities/Wording/ Other/ No commonalities identifiable (several answers accepted)*
Intrusiveness	I feel that this memory is very intrusive in my mind. It comes to my mind even if I don't want it. *From 1 = Not at all to 5 = Very Often*
Feeling of control	I feel that this memory is stronger than me. I cannot control it. *From 1 = Totally disagree to 5 = Totally agree*
**Additional measures—Study 2**	
Auditory vividness	While remembering, I can hear everything in my mind. *From 1 = not at all to 7 = as vivid as if it were happening now*
Visual vividness	While remembering, I can see everything in my mind. *From 1 = not at all to 7 = as vivid as if it were happening now*
Emotional intensity	While remembering, the emotions I feel are intense. *From 1 = not at all to 7 = extremely*
Belief in accuracy	My memory of the event is an accurate reflection of the event as a neutral observer would report it and is not distorted by my beliefs, motives, and expectations. *From 1 = 100% distorted to 7 = 100% accurate*
Centrality	I feel that this event has become a central part of my life story. *From 1 = totally disagree to 7 = totally agree*
Temporal context	While remembering the event, I can identify when it happens in my life, in relation to other events. *From 1 = not at all to 7 = perfectly*
**Scene/content questions**	
Personal location	While remembering, I can identify where I am in relation to the things that I am remembering. *From 1 = not at all to 7 = I know exactly where I am seeing the event from*
Event content	As I remember, I can identify the actions, objects, and/or people that are involved in the memory, though I may not be able to clearly say where they are in relation to each other. *From 1 = not at all to 7 = definitely*
Event layout	As I remember, I can describe where the actions, objects, and/or people are located in the memory. *From 1 = not at all to 7 = as if it were happening now*
Setting name	While remembering, I can identify or name the setting where the memory occurred, although I might not be able to describe it clearly. *From 1 = not at all to 7 = definitely*
Setting layout	While remembering, I experience a scene in which the elements of the setting are located relative to each other in space. *From 1 = not at all spatially organized to 7 = a clear spatial layout*

* *Questions included in the notebook (Study 1)*.

¤ *Likert-scales ranging from 1 to 5*.

£ *Likert-scales ranging from 1 to 7*.

The second step was to complete a more detailed questionnaire about their involuntary memories, later on the same day, supported by the records in the notebook (see Table 2 for details). Participants used a structured memory diary booklet to record responses to a series of questions meant to explore the subjective characteristics of the memory.

##### Voluntary autobiographical memory collection

Immediately after completing the involuntary memory questionnaire, the last step of the procedure consisted of uncovering a cue-word in the diary and then generating a voluntary memory in response to the cue word. The word cues employed were *working, snow, sport, school, happy, book, friends, biking, shirt, family, rain, cinema, traveling, phone*, and *car* (39). Participants then answered the same series of questions as before, but about the voluntary memory they had just retrieved (see Table 2 for details).

Participants received a reminder phone call about the study every 2 weeks until they had completed the diary. This was done to motivate them to continue the study and to verify that participants, those with schizophrenia in particular, were doing well and/or to resolve any difficulties they might experience in relation to the study.

#### Coding

##### Attention

Participants reported what they were doing when an involuntary memory came to mind and whether they were thinking of something else in parallel with their current task. On the basis of these descriptions, attention was scored as focused or diffuse following a previously used coding scheme (1, 33). Attention was scored as diffuse if (1) the person had explicitly written that he or she was bored, tired and, unengaged with his or her current task; (2) if the person had been thinking of something else in parallel with the current task and the content of this thought appeared to be irrelevant to the current task (e.g., daydreaming about vacations while attending a meeting); (3) if the subject had responded “Nothing” to the question “What were you doing?” or mentioned an activity that probably did not demand much attention, for example “I was waiting for the doctor in the waiting room.” Otherwise, attention was scored as focused. Scores were assigned by the experimenter and by an independent judge; inter-rater agreement was good (κ = 0.72) and disagreements were resolved by discussion.

##### Thematic content

Participants' descriptions were used to code the content of involuntary and voluntary memories using seven categories of frequently recalled events (27): (1) major achievements, (2) mundane childhood events, (3) mundane daily life events, (4) missed opportunities, remorse, or events that did not happen, (5) failures or humiliations, (6) traumatic or life threatening events and (7) death-related events. The content of each memory was also rated by the experimenter and by an independent judge, with good inter-reliability (κ = 0.81). Disagreements were resolved by discussion.

### Results

#### Protocol Completion

Only 20 of the 30 patients who began the diary protocol completed it in full. Ten patients gave up the protocol whereas we lost touch with only three control participants. In other words, there were three times as many drop-outs in the patient group as in the control group. The main difficulties and/or complaints reported by the patients were (1) the lack of motivation and autonomy to complete the diary at home over a period of several weeks, (2) their tendency to focus on potentially unpleasant involuntary autobiographical memories and (3) metacognitive impairments that might have affected diary completion. Importantly, none of the individuals with schizophrenia reported discomfort or any increase of psychotic symptoms due to the study.

#### The Conditions of Elicitation of Involuntary Memories

##### Attention

Each participant reported 15 involuntary memories classified in two categories according to their associated attentional state. Patients tended to experience more involuntary memories when their attention was diffuse, compared to controls [62.98% in patient group and 51.75% in control group; *t*_(38)_ = 1.94, *p* = 0.060].

##### Cues

**Figure 1:**
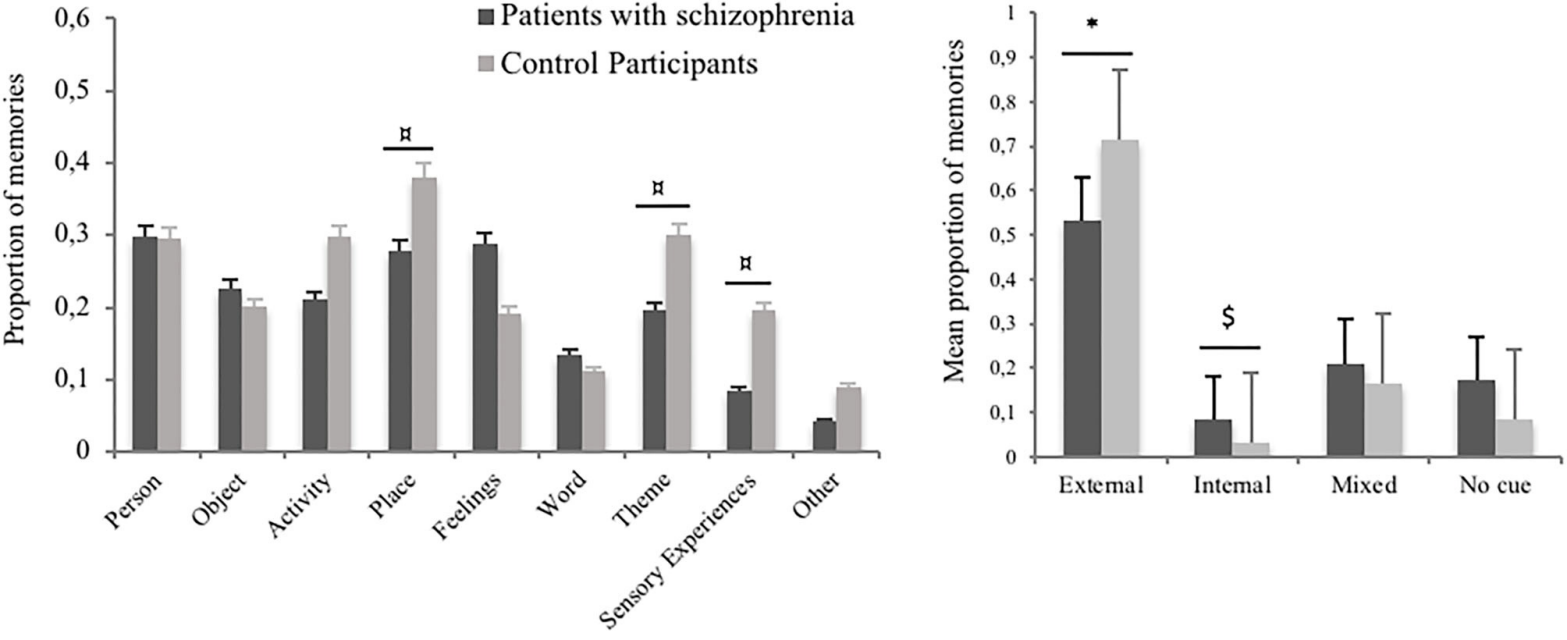
The left plot shows the proportion of involuntary autobiographical memories triggered by each cue category in patients with schizophrenia and in control participants, in Study 1. The right plot shows the proportion of involuntary autobiographical memories triggered by only external cues, only internal cues, mixed cues, or unidentified cues, in Study 1. ^$^*p* < 0.15; ^¤^*p* < 0.10; *p < 0.05.

A single memory could be elicited by several cues. We calculated a mean score for each type of cue, indicating the proportion of memories for which this cue had been endorsed by the participant. These scores were considered in a 2-by-10 analysis of variance with group (patients with schizophrenia vs. controls) as the between-subjects variable and trigger (person; object; activity; place; feelings; word; sensory experiences; theme; none; other) as a within-subjects variable (see Figure 1, left plot). There was no main effect of group, *F*_(1, 38)_ = 0.47; *p* = 0.49; $\eta_{p}^{\text{2}}$ = 0.01, as both groups identified on average the same number of cues per memory. There was a main effect of cue, *F*_(9, 342)_ = 9.93; *p* < 0.001; $\eta_{p}^{\text{2}}$ = 0.21, indicating that overall some cues elicited more frequent involuntary memories (e.g., places, persons) than others (e.g., words). There was also an interaction between group and cue, *F*_(9, 342)_ = 1.98; *p* = 0.04; $\eta_{p}^{\text{2}}$ = 0.05, reflecting some variations in the types of triggers that elicited involuntary autobiographical memories in the two groups. *Post-hoc* analyses showed that the cue categories “place,” “theme,” and “sensory experiences” tended to be less frequent in the patient group in comparison to the control group, *ps* < 0.09.

We compared the proportion of memories triggered by each category of triggers: external cues (i.e., person, object, activity, place, word, sensory experience, and theme), internal cues (i.e., feeling), mixed cues (both external and internal cues), and unidentified cues (memories for which the participant was not able to detect any relationship to his or her present situation) in both groups, using a four-by-two analysis of variance (see Figure 1, right plot). These categories were defined based on participants' triggers identification, controlling for the description of their ongoing thought. We observed a significant effect of the type of cue, *F*_(1, 38)_ = 42.69; *p* < 0.001; $\eta_{p}^{\text{2}}$ = 0.53, but no significant effect of the group, *F*_(1, 38)_ = 1.00; *p* = 32; $\eta_{p}^{\text{2}}$ = 0.03. The interaction between the type of cue and the group was close to significance, *F*_(1, 38)_ = 2.51; *p* = 0.062; $\eta_{p}^{\text{2}}$ = 0.06, reflecting a lower proportion of memories triggered by external cues in the patient group, compared to the control group, *p* = 0.04. Conversely, the proportion of memories triggered by internal cues tended to higher in the patient group than in the control group, *p* = 13.

The comparison, using a two-by-two analysis of variance, of memories triggered by external vs. internal cues, in both groups, showed a significant interaction effect, *F*_(1, 38)_ = 5.13; *p* = 0.03; $\eta_{p}^{\text{2}}$ = 0.12.

#### Characteristics of Involuntary and Voluntary Memories

##### Subjective ratings of involuntary and voluntary autobiographical memories

We compared involuntary and voluntary autobiographical memories in patients with schizophrenia and control participants using a series of two-by-two analyses of variance with group (individuals with schizophrenia vs. control participants) as a between-subjects variable and type of retrieval (involuntary vs. voluntary) as a within-subjects variable (see Table 3). The dependent variables (see Table 2) were mean values, calculated across memories for each participant, or (for the measure of mood impact) the proportion of memories retrieved per participant.

**Table 3:** The characteristics of involuntary and voluntary autobiographical memories in patients with schizophrenia and control participants measured in Study 1.

	**Patients with schizophrenia (*****n*** **=** **25)**				**Control participants (*****n*** **=** **25)**				**Statistics** ***F***_****(1,38)****_					
	**Involuntary**		**Voluntary**		**Involuntary**		**Voluntary**		**Group**		**Retrieval**		**Interaction**	
	***M***	***SD***	***M***	***SD***	***M***	***SD***	***M***	***SD***	***F***	**$\eta_{p}^{2}$**	***F***	**$\eta_{p}^{2}$**	***F***	**$\eta_{p}^{2}$**
**Common measures to Studies 1 & 2**														
Me-ness^*^	4.60	0.57	4.56	0.55	4.79	0.31	4.66	0.53	3.80^¤^	0.09	0.12	0.00	1.40	0.03
Spontaneous rehearsal^*^	2.81	0.88	2.51	0.85	2.90	0.77	2.34	0.61	0.13	0.00	13.47^***^	0.27	0.45	0.01
Visual perspective^*^	2.81	0.99	2.80	1.01	2.86	1.03	2.93	1.08	0.18	0.00	0.54	0.01	0.42	0.01
Belief in occurrence^*^	4.57	0.58	4.56	0.55	4.64	0.53	6.66	0.53	0.83	0.01	0.00	0.00	0.09	0.00
Emotional components	–	–	–	–	–	–	–	–	–	–	–	–	–	–
Emotional valence^*^	3.38	0.62	3.66	0.66	3.51	0.49	3.86	0.48	1.52	0.04	14.79^***^	0.27	0.24	0.01
Negative mood impact^a^	0.20	0.23	0.08	0.15	0.14	0.13	0.08	0.11	0.66	0.02	17.03^***^	0.31	1.15	0.03
No mood impact^a^	0.45	0.30	0.50	0.04	0.54	0.24	0.53	0.21	2.45	0.06	1.04	0.03	2.52	0.06
Positive mood impact^a^	0.34	0.29	0.41	0.04	0.31	0.17	0.39	0.18	1.46	0.04	3.90^¤^	0.09	0.70	0.02
Physical reaction^*^	2.17	0.87	2.18	0.99	2.50	0.81	2.30	0.70	0.23	0.00	1.39	0.03	1.82	0.04
**Additional measures (Study 1)**														
Intrusiveness^*^	1.97	0.86	1.77	0.92	1.97	0.66	1.70	0.63	0.03	0.00	7.42^**^	0.16	0.16	0.00
Feeling of control^*^	2.02	0.88	1.80	0.82	2.02	0.74	1.76	0.75	0.01	0.00	11.24^**^	0.23	0.08	0.00

* *Ratings from 1 to 5*;

a *Proportion of memories*.

¤ *p < 0.10*;

** *p < 0.01*;

*** *p < 0.001*.

We observed several main effects of the type of retrieval. Involuntary memories scored higher on negative mood impact, spontaneous rehearsal, intrusiveness and (lack of) feeling of control, and they were rated lower on (positive) emotional valence than voluntary memories, *Fs*_(1, 38)_ > 7.42; *ps* < 0.01; $\eta_{p}^{\text{2}}$ > 0.16. No significant group effect or interaction effect were observed on any of these subjective ratings, *ps* > 0.13. Only ratings of autobiographical me-ness tended to be lower in the patients group, in comparison to the control group, *F*_(1, 38)_= 3.80; *p* = 0.097; $\eta_{p}^{\text{2}}$ = 0.09.

For both involuntary and voluntary autobiographical memories (respectively, *n* = 512 and *n* = 533), patient and control groups did not differ with regard to the temporal distribution of the memories, χ^2^ < 3.10; *ps* > 0.38.

##### Content of involuntary and voluntary memories

**Figure 2:**
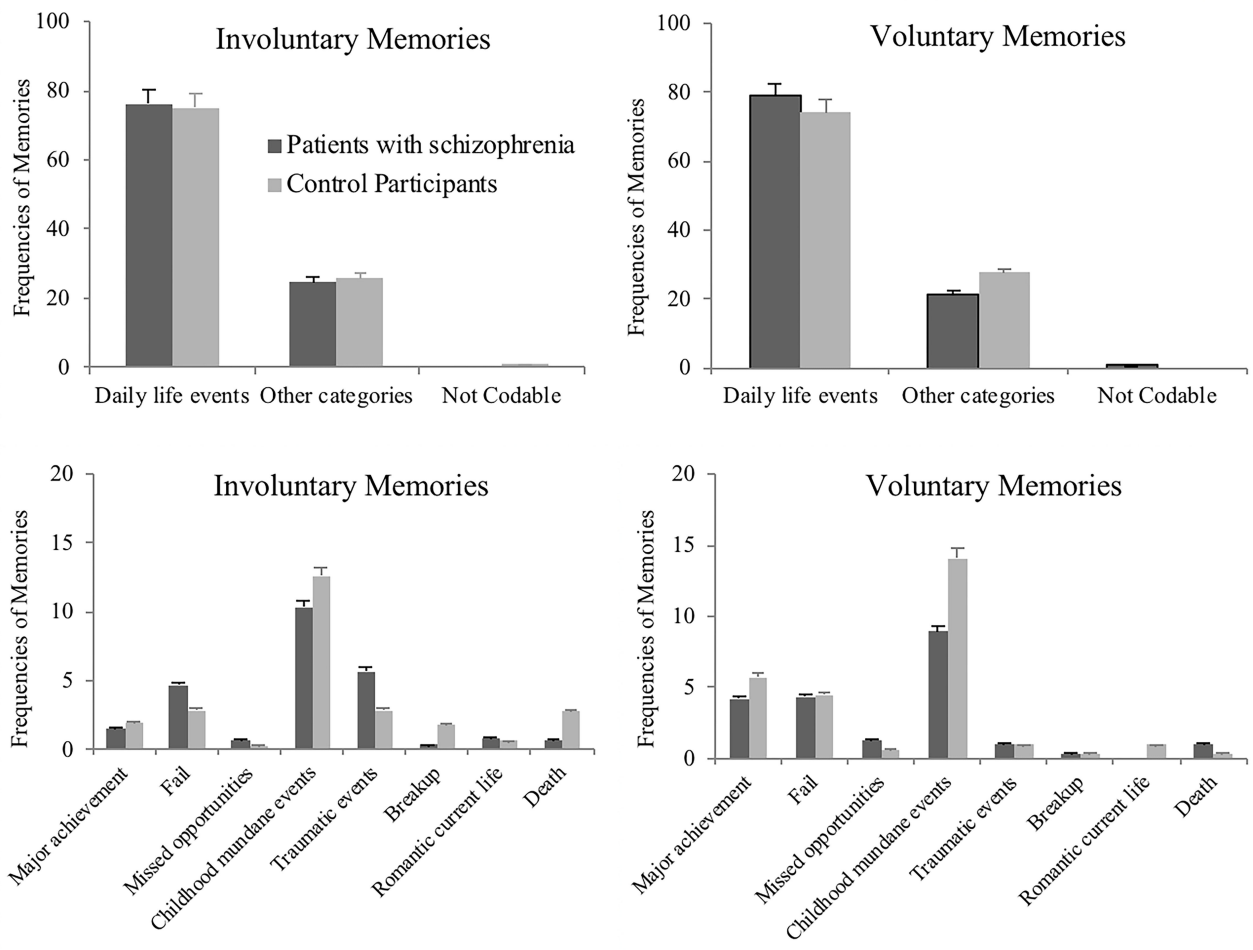
The upper plots show the frequencies of involuntary and voluntary autobiographical memories related to mundane daily life events or other categories of event, in patients with schizophrenia and in control participants, in Study 1. The lower plots show the distribution of these other categories of event, across groups and separately for involuntary and voluntary memories, in Study 1. Error bars represent standard errors.

Consistent with previous studies, a large majority of involuntary and voluntary memories, above 74%, were related to mundane daily life events, in both patient and control groups. Hence, we decided to first compare the proportion of memories related to mundane daily life events to the proportion of memories related to other contents (i.e., all other content categories grouped together), conducting Chi-square analyses separately for involuntary and voluntary memories. The content of both involuntary and voluntary memories did not differ between patients and control participants, respectively, χ^2^ = 2.94; *n* = 526; *p* = 0.23 and χ^2^ = 6.49; *n* = 537; *p* = 0.48 (see Figure 2, upper plots).

Afterwards, a second run of Chi-square analyses were conducted to analyze the distribution of other content categories across groups, for involuntary and voluntary memories separately. The content of autobiographical memories did not differ significantly across groups, for involuntary recall, χ^2^ = 13.12; *n* = 137; *p* = 0.11, or for voluntary recall, χ^2^ = 4.30; *n* = 129; *p* = 0.12 (see Figure 2, lower plots).

### Discussion

The aim of Study 1 was to examine involuntary autobiographical memories of patients with schizophrenia as they spontaneously occur in the course of everyday life. The diary method, used in this study, enabled the collection of critical information about the context in which involuntary memories and spontaneous cognition arise in daily life of people with schizophrenia, which no other method can supply.

With regard to the context in which involuntary autobiographical memories occur, several group differences were identified. First, in comparison to control participants, patients tended to experience more involuntary memories when their attention was diffuse, that is, when not engaged in a current task. This result might be related to those of Elua et al. (18), who showed that patients with schizophrenia, just like control participants, were more likely to report experiencing mind-pops during fairly undemanding activities.

Second, involuntary autobiographical memories were mostly triggered by external environmental cues in both groups, as previously observed (1), but patients reported significantly fewer external cues than controls. Moreover, internal cues (i.e., cues that are only present in thought) tended to trigger involuntary memories more frequently in the patient group, than in the control group. This result might reflect patients' hyper-reflexivity, a symptom related to self-disorders in schizophrenia. The hyper-reflexivity refers to an exaggerated self-consciousness, a (fundamentally non-volitional) tendency to focus on inner processes and phenomena that would normally be experienced tacitly, making them explicit (40, 41).

The present diary study replicated some of the effects of involuntary vs. voluntary retrieval on memory characteristics (for mood impact and spontaneous rehearsal), reported in the literature on non-clinical populations (3, 33, 42). These observations confirm the diary protocol was an effective method for collecting and differentiating between involuntary and voluntary autobiographical memories, in the current sample of individuals with schizophrenia and healthy controls.

Considering all memory measures included in Study 1, only autobiographical me-ness tended to be lower in participants with schizophrenia than in control participants, as previously observed for sensory-cued (voluntary) autobiographical memories (29). However, no other group or interaction effects were observed on memory characteristics self-rated by participants, contrary to what was observed in Allé et al. (30) for contextual information and feeling of reliving. The absence of such commonly observed differences [e.g., (28)] might be due to the high dropout rate in the patient group, leading this group to be overrepresented by well-functioning patients. This bias might have attenuated differences between the memory characteristics reported by patients vs. controls.

Quite surprisingly, neither spontaneous rehearsal, nor intrusiveness, nor feeling of control differed between the schizophrenia and control groups. These results could simply reflect the mundane nature of the reported memories in the current study (as reflected in the content coding). Alternatively, these results may reflect patients' active attempt to avoid memories that could be painful or uncomfortable, or their poorer ability to report the most intrusive memories (considering that they might not be able to adequately distinguish them from hallucinations, due to metacognitive deficits).

Several limitations of Study 1 should be acknowledged. The diary method did not allow any control over the type of thought reported in the diary or the proper completion of the questionnaires. Besides, the diary method might have been taxing for participants, as it requires participants to recall the task instructions and hold the memory in mind while they completed the questionnaires.

Study 2 was undertaken to address limitations of Study 1 and replicate and extend its findings. First, the experimental design of Study 2 reduced the cognitive effort required to report involuntary memories, compared with the diary procedure used in Study 1. The task composing Study 2 was less demanding for participants in terms of motivation and autonomy. Besides, the presence of the experimenter during the task made possible a real-time control of the participants' understanding and completion of the questionnaires. Second, Study 2 also enabled to deepen the investigation of involuntary autobiographical memories in schizophrenia, both in terms of qualitative characteristics (including vividness, centrality, emotional intensity, and spatio-temporal context) and conditions in which they are elicited.

## Study 2

### Methods

#### Participants

Twenty-five outpatients with schizophrenia (seven women and 18 men) from the Psychiatry Department of Strasbourg University Hospitals (France) took part in the study, as well as a group of 25 matched control participants (see Table 1). Patients' and control participants' recruitment was based on the same criteria as in Study 1.

#### Design

The design was a 2 (Group: patients with schizophrenia vs. control participants) ×2 (Retrieval: involuntary vs. voluntary) mixed design with Retrieval as within-subject variables.

#### Materials

A computerized task was designed in E-Prime 2.0 Professional (Psychology Software Tools), consisting of two distinct sessions: the involuntary autobiographical memory elicitation and the voluntary autobiographical memory collection.

Two series of words or phrases, previously recorded by the experimenter and orally presented via a laptop, were used as cues, either to elicit involuntary autobiographical memory in Session 1 or to sample voluntary autobiographical memories in Session 2. Each series of words were composed of 80 items, always presented in the same order, based on words previously used by Berntsen (1) and included various semantic categories (activities, objects, sensory experiences, generic—non personal—themes, life themes, places, wordings, feelings). The two series were randomly assigned to the involuntary vs. voluntary memory sessions.

#### Procedure

Each participant was individually tested at the Psychiatry Department of the University Hospital of Strasbourg, France. The involuntary and voluntary memory sessions took place at 2 different days (separated by a maximum of 7 days) and were always administered in the same order to avoid the contamination of the involuntary autobiographical memory elicitation with the voluntary autobiographical memory collection.

Participants were seated in front of the laptop displaying a black screen. Each series of words or phrases was played aloud.

##### Involuntary autobiographical memory session

During the first session, participants were presented the series of words (also referred as cues in the results section) played every 5 s, and were instructed to do nothing but listening carefully to those words. If, at some point, they thought about a past situation during the task, they had to press the spacebar to interrupt the task. The button press aimed to record retrieval time for each involuntary memory elicited. Immediately after the task interruption, a written description of the memory was collected, and the experimenter made sure that the spontaneous thought reported was an actual memory and not another kind of thoughts (i.e., general statement on his/her life, future projection, prospective memory, etc.), by asking the following question: “Are you reporting a memory of past situation you lived?” The number of false alarms was recorded.

When 10 involuntary autobiographical memories had been elicited, the audio-recorded reading of words was terminated. Hence, the number of cues used to elicit the 10 involuntary autobiographical memories varied according to participants' proneness to experience involuntary memories. Participants self-rated memory characteristics using 7-point Likert scales. Some measures were common to Study 1 and additional measures were included to assess vividness, emotional intensity, or context [questions were derived or modified from previous work; (43–45) see Table 2].

##### Voluntary autobiographical memory session

The second session consisted of exactly the same setup, except that participants were specifically asked to strategically retrieve a memory in response to each word or phrase orally presented. In this condition, the series of words was presented with a 60-s delay between each word, to give participant time to think about a personal event and find a memory. Similar to the first session, participants were told to immediately report when they remembered a past event, by pressing the spacebar. Once 10 voluntary memories were collected, the word reading was stopped and participants were asked to answer the same questions as in the involuntary condition to assess the characteristics of their voluntary autobiographical memories (see Table 2).

### Results

#### Protocol Completion

All participants (patients and controls) fully completed the protocol. There is no dropout to report in Study 2.

#### Experimental Validity: Effects of Voluntary vs. Involuntary Retrieval

We first checked whether the thoughts reported in involuntary and voluntary conditions were actual autobiographical memories. First, in the involuntary condition, the total number of button presses (i.e., task interruptions to report a memory) was recorded. The number of false alarm (i.e., pressing a button to report a thought different from a memory) was compared between both patient and control groups. Patients (M = 2.95; SD = 3.43) committed more false alarms than control participants (M = 1.08; SD = 1.70; *t* = 2.33; *p* = 0.02; *d* = −0.19).

In the voluntary condition, non-autobiographical memories were counted from participants descriptions of their memories, but other thought occurrence was extremely rare (1 for 1 patient and for 2 control participants), meaning that participants successfully completed the voluntary condition of the protocol.

Second, we examined how involuntary retrieval differs from voluntary retrieval (see Table 4 for statistics). In order to validate the new paradigm we developed, we needed to observe robust differences between the two within-subjects conditions.

**Table 4:** Retrieval characteristics of involuntary and voluntary autobiographical memories in patients with schizophrenia and control participants, in Study 2.

	**Patients with schizophrenia (*****n*** **=** **25)**				**Control participants (*****n*** **=** **25)**				**Statistics** ***F***_****(1,48)****_					
	**Involuntary**		**Voluntary**		**Involuntary**		**Voluntary**		**Group**		**Retrieval**		**Interaction**	
	***M***	***SD***	***M***	***SD***	***M***	***SD***	***M***	***SD***	***F***	**$\eta_{p}^{2}$**	***F***	**$\eta_{p}^{2}$**	***F***	**$\eta_{p}^{2}$**
Number of memories	8.37	2.34	9.54	1.18	7.36	2.60	9.92	0.40	0.62	0.01	29.88^***^	0.39	4.18^*^	0.08
Log (retrieval time in seconds)	3.58	0.09	3.99	0.23	3.57	0.07	3.95	0.18	0.89	0.02	145.19^***^	0.76	0.21	0.01
Number of cues	60.21	21.94	12.58	4.82	72.62	15.10	12.50	4.19	4.72^*^	0.09	383.34^***^	0.89	5.16^*^	0.10
Cognitive effort	2.58	1.61	3.72	1.10	1.72	0.68	3.07	1.24	7.22^*^	0.13	40.32^***^	0.46	0.29	0.01
Surprise effect	3.53	1.52	3.90	1.27	4.21	1.70	3.97	1.62	1.06	0.02	0.07	0.00	1.57	0.03

* *p < 0.05*;

*** *p < 0.001*.

First, as expected, the retrieval time was significantly higher in the voluntary condition, strongly supporting the distinction between the two types of retrieval. Second, the number of memories elicited was significantly higher in the voluntary condition compared to the involuntary condition. Third, the number of cues (i.e., words or phrases) necessary to elicit autobiographical memories was significantly higher in the involuntary condition than in the voluntary condition, reflecting that involuntary memories required, on average, five times more cues than voluntary memories. These findings are consistent with previous work on involuntary vs. voluntary memories in healthy individuals, using an experimental approach (46). Participants also assessed their cognitive effort associated with memory retrieval as being higher in the voluntary condition compared to the involuntary condition.

Emotional intensity and spontaneous rehearsal were higher in involuntary autobiographical memories in comparison to voluntary autobiographical memories, *Fs*_(1, 48)_ > 4.21; *ps* < 0.03; $\eta_{p}^{\text{2}}$ > 0.08 (see Table 5), whereas the surprise effect associated with retrieval did not differ between involuntary and voluntary retrieval. In contrast, belief in occurrence was lower in involuntary autobiographical memories in comparison to voluntary autobiographical memories, *F*_(1, 48)_ = 11.14; *p* = 0.01; $\eta_{p}^{\text{2}}$ = 0.20 (see Table 5).

**Table 5:** The characteristics of involuntary and voluntary autobiographical memories in patients with schizophrenia and control participants in Study 2.

	**Patients with schizophrenia (*****n*** **=** **25)**				**Control participants (*****n*** **=** **25)**				**Statistics** ***F***_****(1,48)****_					
	**Involuntary**		**Voluntary**		**Involuntary**		**Voluntary**		**Group**		**Retrieval**		**Interaction**	
	***M***	***SD***	***M***	***SD***	***M***	***SD***	***M***	***SD***	***F***	**$\eta_{p}^{2}$**	***F***	**$\eta_{p}^{2}$**	***F***	**$\eta_{p}^{2}$**
**Common measures to Studies 1 & 2**														
Retention Time (years)	9.52	9.26	12.46	10.29	9.60	7.55	9.87	8.80	0.60	0.01	0.76	0.02	1.35	0.03
Me-ness^*^	6.65	0.65	6.53	0.67	6.86	0.26	6.68	0.67	1.59	0.03	3.02^¤^	0.06	0.12	0.00
Spontaneous Rehearsal^*^	3.16	1.36	2.81	1.07	3.68	1.31	3.25	1.12	3.38^¤^	0.07	6.34^*^	0.12	0.42	0.01
Visual Perspective^*^	2.87	1.14	2.78	1.31	2.67	1.18	2.40	1.25	0.85	0.02	1.67	0.03	0.45	0.01
Belief in Occurrence^*^	6.67	0.65	6.86	0.49	6.91	0.27	6.89	0.37	1.17	0.02	4.21^*^	0.08	6.43^*^	0.12
Emotional components														
Emotional valence^*^	4.35	0.97	4.83	1.69	4.38	1.28	4.35	0.65	0.63	0.01	1.33	0.03	1.75	0.04
Negative mood impact^a^	0.10	0.16	0.13	0.18	0.15	0.18	0.12	0.10	0.28	0.01	0.05	0.00	1.56	0.03
No mood impact^a^	0.62	0.31	0.54	0.34	0.51	0.33	0.59	0.23	0.15	0.00	0.02	0.00	3.94^¤^	0.08
Positive mood impact^a^	0.29	0.26	0.32	0.23	0.34	0.30	0.28	0.21	0.01	0.00	0.18	0.00	1.62	0.03
Physical reaction^*^	2.50	1.45	2.39	1.36	3.13	1.67	2.81	1.24	3.81^¤^	0.08	1.15	0.03	0.43	0.01
**Additional measures (Study 2)**														
Auditory vividness^*^	3.87	1.45	4.08	1.40	5.37	0.93	5.18	1.16	16.38^***^	0.26	0.00	0.00	1.70	0.03
Visual vividness^*^	5.19	0.9	5.12	0.81	5.95	0.67	5.96	0.60	16.74^***^	0.26	0.11	0.00	0.16	0.00
Emotional intensity^*^	3.80	1.30	3.62	1.34	4.22	1.33	3.44	1.00	0.14	0.00	11.14^**^	0.20	4.18^*^	0.08
Belief in accuracy^*^	6.61	0.66	6.65	0.59	6.88	0.37	6.79	0.56	1.9	0.04	0.19	0.00	1.14	0.02
Centrality^*^	3.39	1.45	3.48	1.32	2.66	1.16	2.68	1.17	5.48^*^	0.10	0.11	0.00	0.45	0.00
Temporal context^*^	5.19	1.04	5.14	1.14	5.93	0.89	5.77	0.71	7.92^**^	0.14	0.74	0.02	0.18	0.00
Scene/content questions														
Personal location^*^	5.33	0.86	5.13	0.88	5.70	0.87	5.67	0.88	4.43^*^	0.09	0.86	0.02	0.51	0.01
Event content^*^	5.00	0.99	4.76	1.04	5.64	0.85	5.61	0.77	10.89^**^	0.19	1.05	0.02	0.54	0.01
Event layout^*^	4.63	1.10	4.37	1.06	5.30	0.89	5.36	0.86	10.29^**^	0.18	0.74	0.01	1.90	0.04
Setting name^*^	5.50	1.08	5.32	0.95	5.77	0.79	5.63	0.92	1.35	0.03	2.51	0.05	0.04	0.00
Setting layout^*^	5.03	1.12	5.03	1.01	5.35	0.95	5.48	1.03	1.90	0.04	0.38	0.01	0.41	0.01

* *Ratings from 1 to 7*;

a *Proportion of memories*.

¤ *p < 0.10*;

** *p < 0.01*;

*** *p < 0.001*.

Overall, these findings support the validity of the current experimental paradigm by showing that this paradigm reliably distinguished involuntary from voluntary memories.

#### Effects of Patient vs. Control Group and Interaction Effects

##### The conditions of elicitation of involuntary memories (see Figure 3)

**Figure 3:**
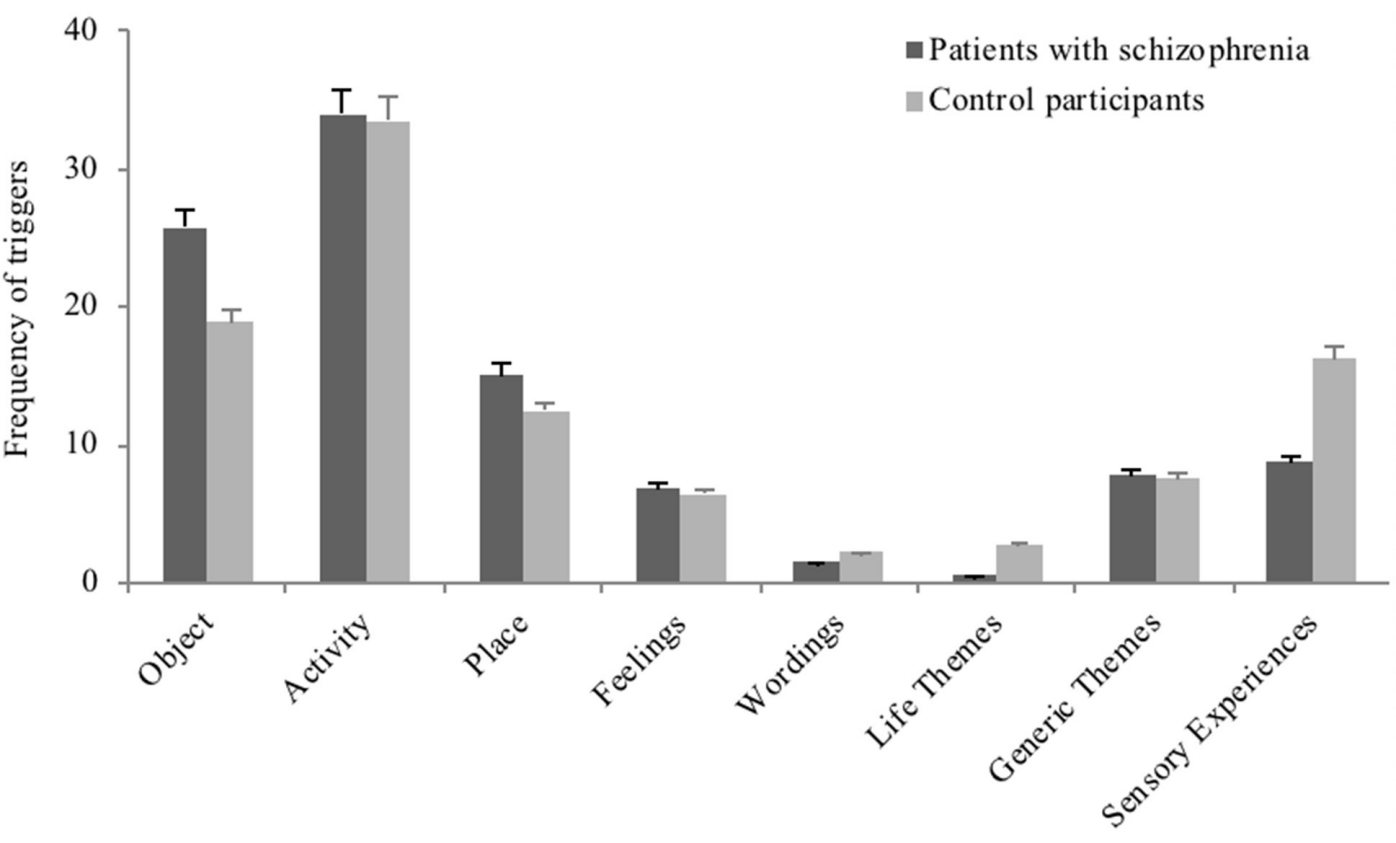
The plot shows the proportion of involuntary autobiographical memories triggered by each cue category in patients with schizophrenia and in control participants, in Study 2. Error bars represent standard errors.

The category of memory triggers (i.e., activities, objects, sensory experiences, generic—non-personal—themes, life themes, places, wordings or feelings) did not differ between patient and control groups, χ^2^ = 10.35; *n* = 391; *p* = 0.17 (see Figure 3).

##### Self-rated qualitative characteristics of autobiographical memory

We compared involuntary and voluntary autobiographical memories in individuals with schizophrenia and control participants using a series of two-by-two analyses of variance with group (schizophrenia vs. control) as a between-subjects variable and type of retrieval (involuntary vs. voluntary) as a within-subjects variable (see Table 5). The dependent variables were mean values, calculated across memories for each participant, or (for mood impact) the proportion of memories retrieved per participant.

However, we observed several main effects of group, showing a pattern of lower ratings in the patient group, in comparison to the control group, for auditory and visual vividness, *Fs*_(1, 48)_ > 16.38; *ps* < 0.001; $\eta_{p}^{\text{2}}$ > 0.26, temporal context, *F*_(1, 48)_ = 7.92; *p* = 0.007; $\eta_{p}^{\text{2}}$ = 0.14, personal location, *F*_(1, 48)_ = 4.43; *p* = 0.04; $\eta_{p}^{\text{2}}$ = 0.09, event content, *F*_(1, 48)_ = 10.89; *p* = 0.002; $\eta_{p}^{\text{2}}$ = 0.19, and event layout, *F*_(1, 48)_ = 10.29; *p* = 0.002; $\eta_{p}^{\text{2}}$ = 0.18. Trends were also observed for the frequency of spontaneous rehearsal and the visual perspective, *Fs*_(1, 48)_ > 3.38; *ps* < 0.07; $\eta_{p}^{\text{2}}$ > 0.07. Centrality of events was rated higher in patient group in comparison to control group, *F*_(1, 48)_ = 5.48; *p* = 0.02; $\eta_{p}^{\text{2}}$ = 0.10.

Two significant interactions were observed between group and type of retrieval for belief in occurrence and emotional intensity, *Fs*_(1, 48)_ > 4.18; *ps* < 0.04; $\eta_{p}^{\text{2}}$ > 0.08. *Post-hoc* analyses showed that patients' involuntary memories were rated as emotionally less intense than controls' involuntary memories, *p* < 0.001, whereas no difference was observed for voluntary memories. The belief in occurrence was significantly lower for patients' involuntary autobiographical memories in comparison to their voluntary memories, *p* = 0.002, whereas this difference was not present in the control group.

In addition, a trend was observed for the proportion of memories with no mood impact, *F*_(1, 48)_ = 3.94; *p* = 0.053; $\eta_{p}^{\text{2}}$ = 0.08, reflecting that in the patient group the proportion of memories with no mood impact was higher in the involuntary condition in comparison to the voluntary condition, while the reverse pattern being observed in the control group.

No group effect was observed on the retention time of the autobiographical memories, *F*_(1, 48)_ = 0.60; *p* = 0.44; $\eta_{p}^{\text{2}}$ = 0.01.

##### Content of involuntary and voluntary memories

**Figure 4:**
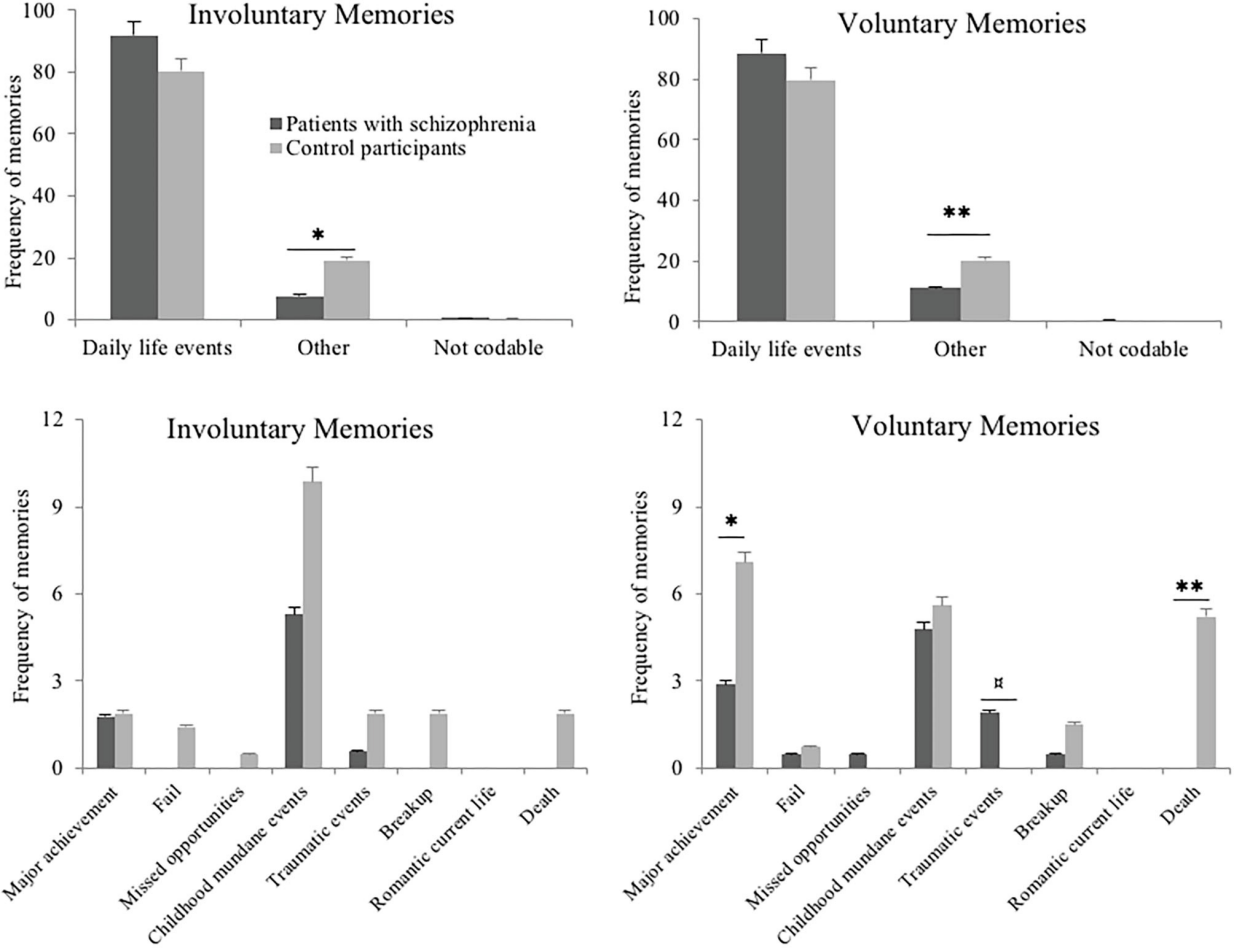
The upper plots show the frequencies of involuntary and voluntary autobiographical memories related to mundane daily life events or other categories of event, in patients with schizophrenia and in control participants, in Study 2. The lower plots show the distribution of these other categories of event, across groups and separately for involuntary and voluntary memories, in Study 2. Error bars represent standard errors. ^¤^*p* < 0.10; *p < 0.05; **p ≤ 0.01.

Similar to Study 1, we first compared the proportion of memories related to mundane daily life events to the proportion of memories related to other contents (i.e., all other content categories grouped together), conducting Chi-square analyses separately for involuntary and voluntary autobiographical memories. The content of both involuntary and voluntary memories differed between patients and control participants, respectively, χ^2^ = 10.49; *n* = 383; *p* = 0.001 and χ^2^ = 7.14; *n* = 487; *p* = 0.007 (see Figure 4, upper plots), reflecting that patients recorded more memories classified as “other.”

Afterwards, a series of Chi-square analyses was conducted to analyze the distribution of other content categories across groups, for involuntary and voluntary memories separately (respectively, *n* = 54 and *n* = 77).

With regard to involuntary autobiographical memory, the thematic content of memories was highly similar between patients and control participants, a comparison across all event categories using Fisher's exact test showed do significant differences (all *ps* > 0.10)

On contrary, the thematic content of voluntary autobiographical memories differed between patient and control groups for “major achievement” category, χ^2^ = 4.16; *n* = 25; *p* = 0.04 and “death” category, χ^2^ = 9.33; *n* = 14; *p* = 0.002, which were less frequently observed in patients' memories than in controls' memories. The other categories of thematic content were similarly represented across groups; comparisons across all event categories using Fisher's exact test showed do significant differences (all *ps* > 0.10).

### Discussion

Study 2 was conducted to further pursue the mechanisms underlying the activation of involuntary memories and to explore other qualitative characteristics of involuntary memories in schizophrenia, using an experimental paradigm. Importantly, the new experimental set-up showed to be efficient in eliciting involuntary memories and distinguishing them from voluntary memories, and thereby provided a methodological advancement to address limitations observed in Study 1.

Study 2 replicated and extended the findings of Study 1. First, similarly to Study 1 results, Study 2 showed no significant group differences on me-ness, spontaneous rehearsal, visual perspective, emotional component, and on memories content—the majority referring to mundane, daily life events, in both patient and control groups.

Second, with regard to the mechanisms of memories activation, the experimental setup of Study 2 allowed us to observe that patients with schizophrenia reported involuntary autobiographical memories more frequently than control participants, using overall fewer cues. In other words, involuntary autobiographical memories, but not their voluntary counterpart, were more easily triggered in the patient group, compared to the control group. This may reflect patients' sensitivity to triggers, and their specific proneness to involuntary autobiographical memories. These results can be related to those of Study 1 showing that patients had difficulties identifying memories' triggers, probably reflecting an overwhelming sensitivity to triggers.

In addition to their involuntary memory proneness, patients with schizophrenia reported significantly more other types of thoughts in the involuntary condition, compared to controls and to the voluntary condition. This finding likely reflects patients' difficulties in scanning and monitoring their stream of consciousness in order to report only involuntary memories (and not spontaneous future projections, personal reflections or prospective memories, for instance), as instructed. This relates to patients' metacognitive deficits (47–50) or internal source-monitoring deficits (51), that is, their poorer ability to distinguish between different internal sources of mental representation, such as memories from imagined events (52, 53).

These results are in agreement with research on thought disorders in schizophrenia, showing higher semantic association (54, 55), more frequent cognitive intrusions (16), and more frequent semantic mind-pops (17, 18) in schizophrenia patients, in comparison to control groups.

Going further, the new measures added to Study 2 showed significant group differences, reflecting that patients' memories (both involuntary and voluntary) were less vivid, had less spatial and temporal context information, but were rated as more central to identity than those of controls. No interaction effects were observed on those variables, meaning that involuntary and voluntary autobiographical memories were similarly impaired in terms of vividness and scene measures, but enhanced with regard to their centrality. The findings on impaired vividness and contextual information are in full agreement with existing literature on voluntary [e.g., (28, 34, 35)] and involuntary (30) autobiographical memory in schizophrenia.

Nonetheless, the higher centrality of autobiographical memory in the patient group contradicts previous results showing no differences between patients and control participants regarding centrality for very important personal events (56), or studies showing a deficit in self-integration of important autobiographical memories in schizophrenia (56–58). Surprisingly, in the current study, while most of the reported memories were related to mundane events in both groups (see content coding), and not particularly emotional, patients attributed a higher degree of centrality to their memories in comparison to control participants. This finding is consistent with an enhanced self-focus observed in most clinical disorders (59). In addition, the higher centrality of autobiographical memories in the patient group, in spite of their mundane content, could relate to the assignment of inappropriate salience usually observed in schizophrenia, leading certain precepts and ideas to be assigned an exaggerated importance [for a review see (60)]. The aberrant salience to non-relevant stimuli has been thought to relate to difficulties in differentiating self-relevant from self-irrelevant information, which might be explained by dysfunctional self-referential processes (61). Therefore, impairment in self-referential processes in schizophrenia might explain the higher centrality of patients' memories in comparison to controls.

Two significant interactions between group and type of retrieval were observed in Study 2. First, involuntary autobiographical memories were emotionally more intense than their voluntary counterpart in the control group, which is in line with some previous findings (62, 63), whereas this difference was not significant in the patient group.

Second, a significant interaction was also observed for belief in occurrence, showing that patients' involuntary autobiographical memories were associated with a lower belief in occurrence in comparison to their voluntary memories, whereas no differences were observed in the control group. Because involuntary memories are unintentional and uncontrolled, information about the cognitive operations necessary to reconstruct these memories are lacking, potentially making them less easily recognized as self-generated memories (64). In addition, patients' internal source monitoring deficit affects their ability to distinguish reality (e.g., memories of past events) from imagination (e.g., future projection), which might impact even more severely their involuntary autobiographical memories, compared to voluntary autobiographical memories.

#### General Discussion

Studies 1 and 2 were undertaken to explore involuntary remembering in schizophrenia and the conditions in which it occurs in those patients, both in daily life and in an experimental context. Studies 1 and 2 assessed similar characteristics of autobiographical memories but were also designed to investigate different aspects of involuntary remembering in schizophrenia. Study 1 provided information on involuntary remembering in patients' daily life, while Study 2 allowed us to measure quantitative parameters of involuntary retrieval, not accessible with a diary method (e.g., the retrieval time or the propensity to experience involuntary memories in response to experimenter generated cues).

Studies 1 and 2 provided a body of convergent evidence on the mechanisms underlying the activation of involuntary autobiographical memories in schizophrenia. Patients with schizophrenia were more prone to experience involuntary autobiographical memories and more sensitive to triggers (Study 2). In particular, they seemed to be more sensitive to internal cues than controls (Study 1). The higher proneness to experiencing involuntary autobiographical memory in patients might be caused by deficits in intentional inhibition, which are well-described in schizophrenia (65, 66), making spontaneous memories difficult to control and avoid.

The findings from both studies might be related to patients' impairments in self-reflective processes (including metacognition, self-referential, and internal source-monitoring). More precisely, patients' difficulties with identifying their involuntary memories *per se* (Study 2) are likely to be caused by patients' metacognitive deficits (47–50). The lower proportion of external cues and higher proportion of internal cues in the patient group, compared to the control group in Study 1, might be related to an aberrant salience of inner stimuli (40, 41). Relatedly, despite the mundane nature of both patients and controls' involuntary memories, patients rated their memories as being more central to their identity in comparison to controls, thus allocating a higher salience to their mental events. Together, these findings might reflect deficits in self-referential processes in schizophrenia. Finally, patients' involuntary autobiographical memories were associated with a lower belief in occurrence, compared to their voluntary memories and compared to both involuntary and voluntary memories in the control group, possibly reflecting source monitoring disorders in schizophrenia. All together, these results suggest that self-disorders may alter how patients involuntarily remember personal events and the associated experience of control.

To sum up, the difficulty of patients in identifying their mental states and thoughts, associated with a lack of cognitive control, could make them more sensitive to triggers (in particular internal triggers) that elicit involuntary autobiographical memories. Hence, patients' involuntary memories, mostly related to mundane events with low emotional load, are experienced more frequently. Besides, although involuntary and voluntary memories were vague, poorly contextualized and associated with a lower belief in occurrence, patients considered them as more central to the self.

Recent works highlighted the relationship between involuntary autobiographical memory and hallucination within the psychosis continuum including people with schizophrenia (26, 27). Hallucinations are characterized by a failure of self-recognition leading to the externalization of self-generated events [for a review see (67)]. Among the various candidates thought to explain hallucinations (inner speech, semantic mind-pops, memory, etc.), memories of personal events have the potential to account for the wide phenomenological diversity of hallucinations in schizophrenia as ~60% of patients also report non-verbal hallucinations (i.e., not voices), such as environmental noises or animal sounds, in addition to regular verbal hallucinations (68, 69). Moreover, a majority of patients report to know the identity of their voices, and about half agree with the idea that the content of their voices could be the reproduction of speech that they have heard in the past (70).

Although indirectly and speculatively, the present studies on involuntary remembering in patients with schizophrenia provided new evidence suggesting parallels between hallucination and involuntary autobiographical memories [see also (26)]. Concerning the conditions in which involuntary memories occur, we showed that, similarly to hallucinations (68, 71), they were more likely to pop-up during diffuse attentional states (Study 1). Besides, we observed that patients' involuntary autobiographical remembering was also characterized by a deficit of self-reflective processes (impaired triggers identification and more internal triggers in Study 1, and impaired involuntary autobiographical memories identification, higher centrality and lower belief in occurrence in Study 2).

With regard to the emotional component of autobiographical memory, results were mixed. No differences were observed between groups, in terms of emotional valence or mood impact, in any of the two studies. However, in Study 2, patients' memories tended to be less associated with physical reaction or mood impact, than did memories of controls, but these tendencies were not seen in Study 1. Finally, emotional intensity was similar in patients' involuntary and voluntary autobiographical memories, whereas the control group's involuntary autobiographical memories were more emotionally intense, in comparison to their voluntary autobiographical memories. This result differs from previous findings, showing higher emotional intensity associated with involuntary autobiographical memories of individuals with attenuated psychotic symptoms (26, 27) and also higher proportion of trauma-related involuntary memories in the psychosis continuum (26). However, these prior studies focused only on examples of selected involuntary autobiographical memories that were retrospectively rated, which may have led to a biased collection of salient involuntary memories. On the contrary, words or phrases describing everyday life activities and objects were used in the present study to trigger involuntary memories, leading the memories to mostly refer to mundane events.

These results agree with the mixed findings in the literature on emotion and autobiographical memory in schizophrenia and the difficulty to draw any firm conclusions with regard to the emotional component of memory in schizophrenia [see (56, 57, 72–75)]. Future research on emotion and autobiographical memory in schizophrenia could benefit from the use of objective measures of physiological arousal [e.g., (76)] in patients, as these measures have been shown to correlate to some extent with the subjective ratings of emotion in the general population (77).

Studies 1 and 2 both share the limitation that only self-reports were used to assess the qualitative characteristics of autobiographical memories. Insofar as it is well-known that patients also suffer from several impairments of awareness of their own state and of insight [for a review see (78)], the question of patients' abilities to correctly self-assess and report their mental experiences therefore remains. However, in the present studies, we were primarily interested in the contrast of the subjective experience of patients while they were having involuntary vs. voluntary memories. Hence, capturing the subjective experience, regardless of its objective accuracy, is of prime importance with regard to patients' clinical symptoms, such as hallucinations. As a perspective, future research should look at correlations between autobiographical memory measures and cognitive functions in larger sample of participants.

In addition, the design of the present studies might not have allowed us to capture distressing involuntary memories in patients with schizophrenia whereas intrusive thoughts are well-described in literature on thought disorders in schizophrenia (15, 16). Further studies might specifically address stress-related involuntary memories in schizophrenia.

In conclusion, the present studies investigated involuntary remembering in schizophrenia using both a structured diary methodology in participants' everyday environments and in an experimental method in a laboratory context. The results demonstrated that both the conditions under which involuntary memories occur, and the involuntary remembering experience itself, are altered in schizophrenia. Hence, involuntary autobiographical memory presents a specific pattern of alterations in schizophrenia. Viewed together, the findings suggest some parallels between involuntary remembering in schizophrenia and patients' hallucination. Future research should investigate the nature of this relationship, focusing more specifically on distressing involuntary autobiographical memories.

## Data Availability Statement

The raw data supporting the conclusions of this article will be made available by the authors, without undue reservation.

## Ethics Statement

The studies involving human participants were reviewed and approved by Comité de Protection des Personnes Sud Méditerranée IV. Written informed consent to participate in this study was provided by the participants, and where applicable, the participants' legal guardian/next of kin.

## Author Contributions

MA and DB designed the studies. MA collected the data, conducted the analyses, and wrote the first draft of the manuscript. DB, FB, and J-MD provided resources to conduct the research and provided substantial contributions to the manuscript. DB and FB supervised the study. All authors read and approved the final version of the manuscript.

## Conflict of Interest

The authors declare that the research was conducted in the absence of any commercial or financial relationships that could be construed as a potential conflict of interest.
